# Supplementary figures and images for: Physiological, biochemical and phytohormone responses of Elymus nutans to α-pinene-induced allelopathy
Source: PeerJ. 2022 Sep 29;10:e14100. doi: 10.7717/peerj.14100 (PMC9527024; doi:10.7717/peerj.14100)

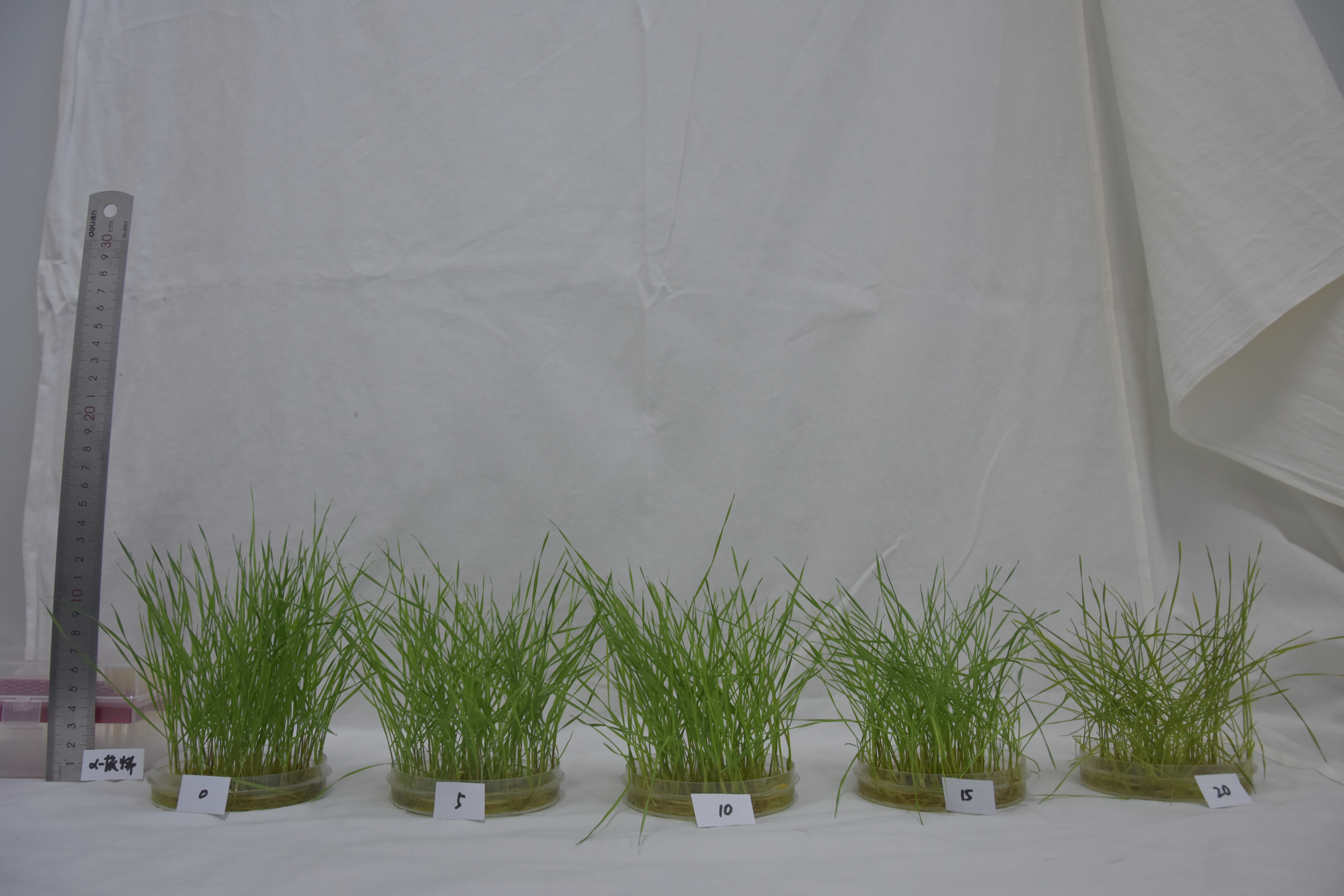

Supplement: Figure S1 [file peerj-10-14100-s002.jpg]

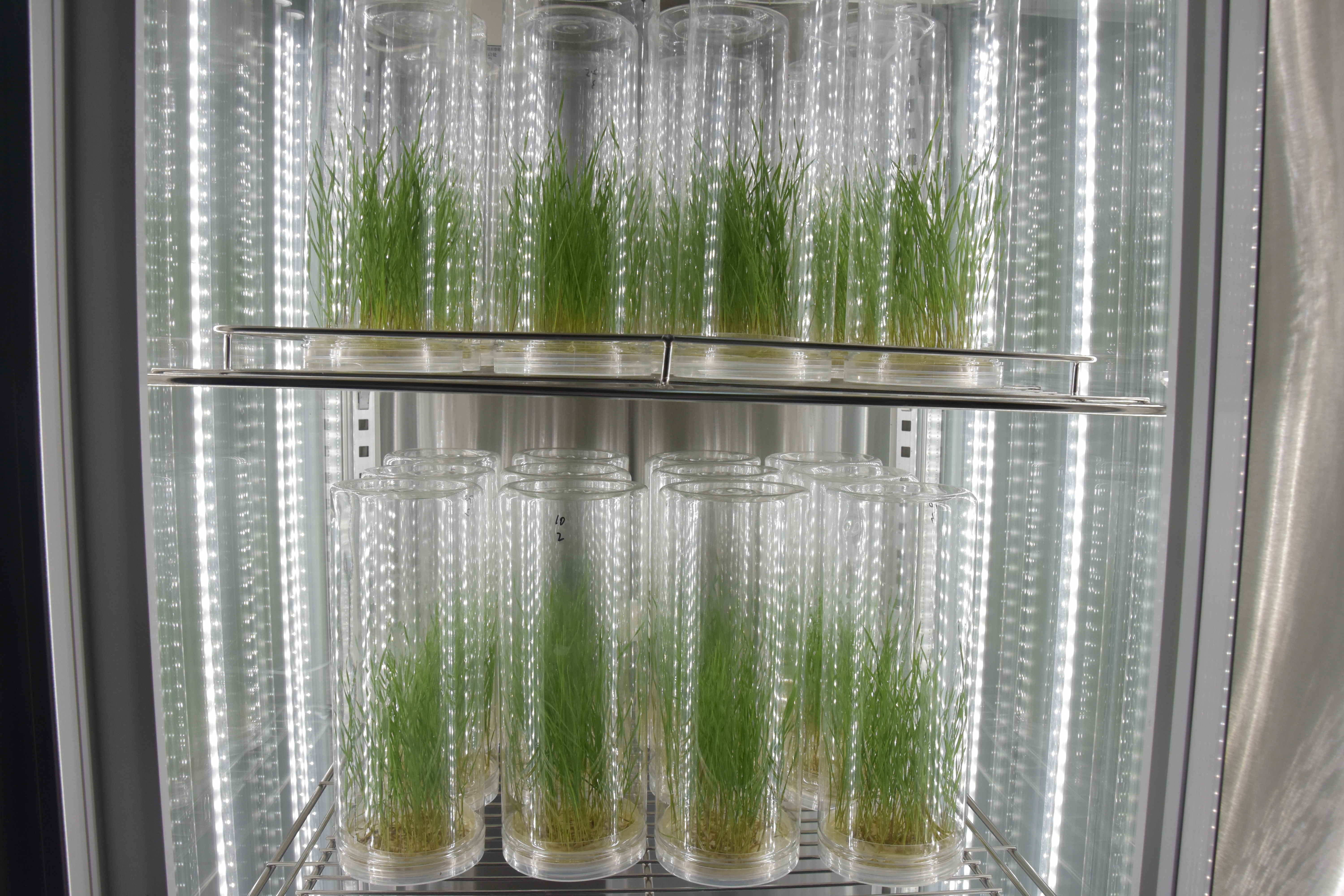

Supplement: Supplemental Information 3 [file peerj-10-14100-s003.jpg]
